# New treatment option for ovarian cancer: PARP inhibitors

**DOI:** 10.1186/s40661-016-0024-7

**Published:** 2016-02-26

**Authors:** Robert S. Meehan, Alice P. Chen

**Affiliations:** Early Clinical Trials Development Program Division of Cancer Treatment and Diagnosis (DCTD), National Institutes of Health (NIH) National Cancer Institute (NCI), 10 Center Drive, Bldg 31, 3A44, Bethesda, MD 20892 USA

**Keywords:** PARP inhibitor, Synthetic lethality, *BRCA*, Homologous recombinant pathway, Base excisional repair pathway

## Abstract

Poly(ADP-ribose) polymerase (PARP), which was first described over 50 years ago by Mandel, are a family of protein enzymes involved in DNA damage response and works by recognizing the single-strand DNA break (ssDNA) and then effecting DNA repair. A double-strand DNA (dsDNA) break can be repaired by one of two different pathways: homologous recombination (HR) or non-homologous end joining (NHEJ). Homologous recombination occurs in the G2 or M phase of the cell cycle when a sister chromatid is available to use as a template for repair. Because a template is available, HR is a high fidelity, error-free form of DNA repair. With NHEJ there is not a template and the DNA is trimmed and ligated which is a very error-prone process of repair which can lead to genetic instability. Exploiting these mechanism led to development of PARP inhibitors with the idea of utilizing synthetic lethality, where two deficiencies each having no effect on the cellular outcome become lethal when combined, as single agent in *BRCA* deficient patients or as chemotherapy/radiotherapy combinations to inhibit ssDNA repair. The recent approval of olaparib in BRCA deficient ovarian cancer patients in US and Europe has opened up a whole new treatment option for ovarian cancer patients. This review will discuss the different PARP inhibitors in development and the potential use of this class of agents in the future.

## Background

Ovarian cancer is the leading cause of death from gynecological malignancies in the United States with an incidence rate of approximately 22,000 and 14,000 deaths per year. Despite all of the headway made in cancer overall, with a risk of dying from cancer decreasing by 20 % since 1991, the relative 5-years survival rates of ovarian cancer has remained poor 36 % in 1970’s and still only 44 % in 2000’s and much worse in late stage disease [1].

Cellular replication is a complex process which is the way living organisms are able to grow and propagate. Replication is a very controlled process with many points of error detection and redundancy to ensure that a high fidelity functioning copy of genetic material is maintained. Essential to this process is the unwinding of the DNA from histone complexes and followed by the active replication processes during S-phase, during this time period DNA is very susceptible to environmental damage or even errors in the replicative process itself [2]. There are a host of detection and repair mechanisms in place which try to minimize errors, which lead to mutations. The BRCA genes are a family of tumor suppressor genes responsible for helping to protect the genome, and the most widely known and studied with current clinical importance are *BRCA1* and *BRCA2* [3]. *BRCA1* is located on chromosome 17 and has many cellular functions such as DNA repair, transcriptional regulation and chromatin remodeling and BRCA2 is located on chromosome 13 and is responsible for DNA recombination and repair primarily by chaperoning RAD51, the enzyme responsible for facilitating recombination [4]. These two genes were described in 1994 and 1995 and the repair pathways which they work have become clinical targets for molecular therapies [5, 6]. Deficiencies in these genes have been historically associated with hereditary breast and ovarian cancer but they also increase risk for uterine, cervical, colon, male breast, prostate, pancreatic cancers, and melanoma [7].

**Fig. 1:**
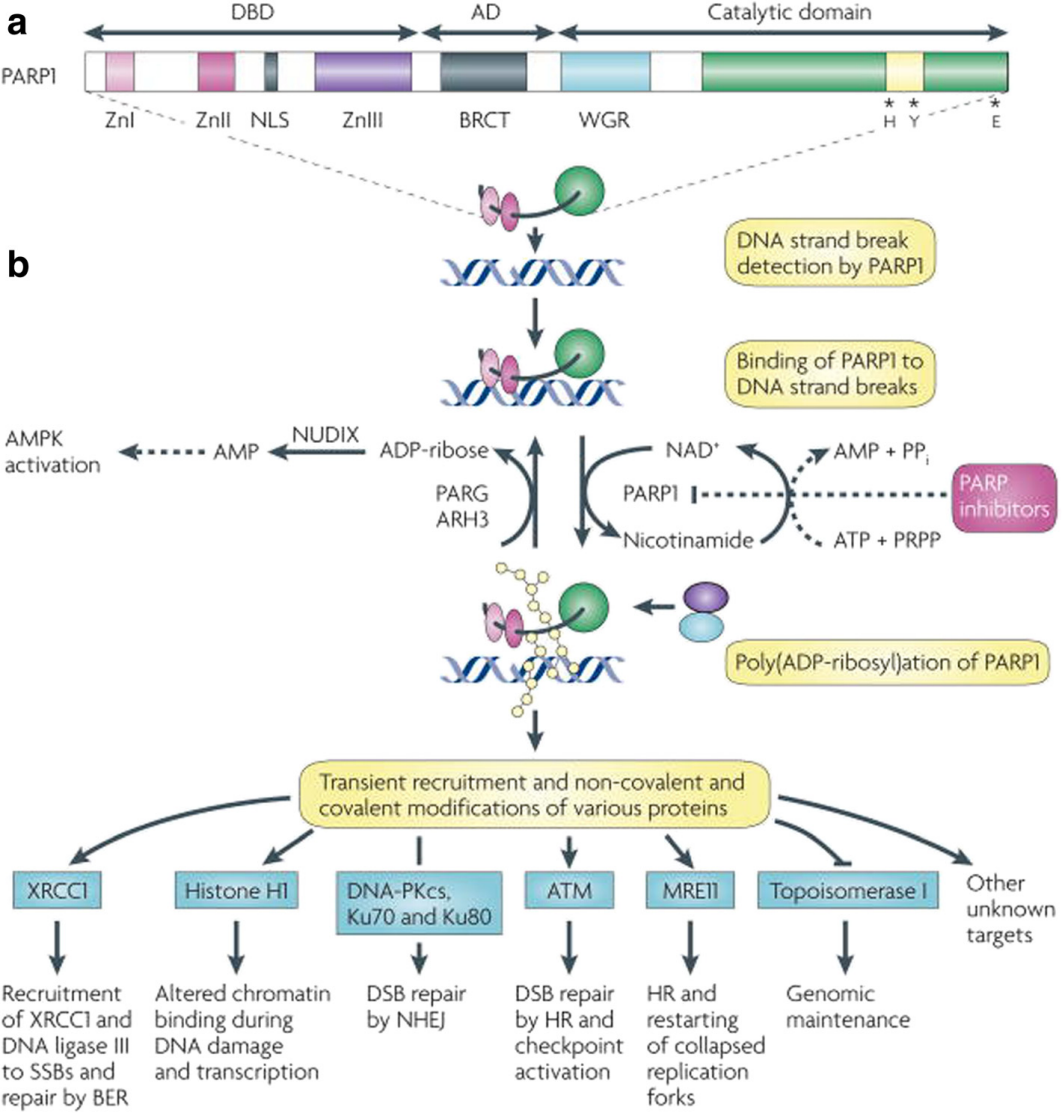
Mechanism of PARP. **a** Poly(ADP-ribose) polymerase 1 (PARP1) is shown with its DNA-binding (DBD), automodification (AD) and catalytic domains. The PARP signature sequence (*yellow box* within the catalytic domain) comprises the sequence most conserved among PARPs. Crucial residues for nicotinamide adenine dinucleotide (NAD^+^) binding (histidine; H and tyrosine; Y) and for polymerase activity (glutamic acid; E) are indicated. **b** | Consequences of PARP1 activation by DNA damage. Although not shown to simplify the scheme, PARP1 is active in a homodimeric form. PARP1 detects DNA damage through its DBD. This activates PARP1 to synthesize poly(ADP) ribose (pADPr; *yellow beads*) on acceptor proteins, including histones and PARP1. Owing to the dense negative charge of pADPr, PARP1 loses affinity for DNA, allowing the recruitment of repair proteins by pADPr to the damaged DNA (*blue* and *purple circles*). Poly(ADP-ribose) glycohydrolase (PARG) and possibly ADP-ribose hydrolase 3 (ARH3) hydrolyse pADPr into ADP-ribose molecules and free pADPr. ADP-ribose is further metabolized by the pyrophosphohydrolase NUDIX enzymes into AMP, raising AMP:ATP ratios, which in turn activate the metabolic sensor AMP-activated protein kinase (AMPK). NAD^+^ is replenished by the enzymatic conversion of nicotinamide into NAD^+^ at the expense of phosphoribosylpyrophosphate (PRPP) and ATP. Examples of proteins non-covalently (pADPr-binding proteins) or covalently poly(ADP-ribosyl)ated are shown with the functional consequences of modification. It is important to note that many potential protein acceptors of pADPr remain to be identified owing to the difficulty of purifying pADPr-binding proteins in vivo. PARP inhibitors prevent the synthesis of pADPr and hinder subsequent downstream repair processes, lengthening the lifetime of DNA lesions. ATM, ataxia telangiectasia-mutated; BER, base excision repair; BRCT, BRCA1 carboxy-terminal repeat motif; DNA-PKcs, DNA-protein kinase catalytic subunit; DSB, double-strand break; HR, homologous recombination; NHEJ, non-homologous end joining; NLS, nuclear localization signal; PP_i_, inorganic pyrophosphate; SSB, single-strand break; Zn, zinc finger. Reprinted by permission from Macmillan Publishers Ltd: Nat Rev Cancer, 2010,10(4):293–301, copyright (2010) [10]

Poly(ADP-ribose) polymerase (PARP), which was first described over 50 years ago by Mandel [8], works by recognizing the single-strand DNA break (ssDNA) and then effecting DNA repair [9] through the base excisional pathway (BER). The proteins consist of two ribose moieties and two phosphates (Fig. 1), and DNA strand breaks are responsible for activating the protein [10]. The PARP catalytic domain binds NAD+ via a unique protein fold, PARP-1 has a combination of zinc fingers and PARP-2 and PARP-3 have different N-terminal domains with very specific regulatory functions in mitotic segregation as well as basal metabolism [11, 12]. PARP is also involved in methylation and transcription of genes coding for cell cycle and stress response, including *p53*. PARP attaches DNA polymerase β to the DNA break site to replace the missing bases [10].

A double-strand DNA (dsDNA) break can be repaired by one of two different pathways: homologous recombination (HR) or non-homologous end joining (NHEJ). Homologous recombination occurs in the G2 or M phase of the cell cycle when a sister chromatid is available to use as a template for repair [13]. Because a template is available, HR is a high fidelity, error-free form of DNA repair. With NHEJ there is not a template and the DNA is trimmed and ligated which is a very error-prone process of repair which can lead to genetic instability [14]. In patients that have *BRCA* deficient HR pathway the BER rescues the cell and leads to a viable cell [15]. When PARP is inhibited in a HR deficient cell, e.g. *BRCA* mutation, the ssDNA break is not repaired by either the BER or HR pathway. [16]. Solid tumors carrying various DNA repair defects have shown increased sensitivity to PARP inhibitors or DNA-damaging chemotherapies [17]. PARP inhibitors have shown activity as monotherapy in cells deficient for the repair of dsDNA breaks by HR as in case of *BRCA* deleterious mutation cells showcasing the principle of synthetic lethality. The concept of synthetic lethality is where two deficiencies each having no effect on the cellular outcome become lethal when combined. Cells which are BRCA deficient and then undergo PARP inhibition, leads to cell death [4].

PARP trapping is another recently described mechanism by which PARP inhibitors are able to kill cancer cells. PARP inhibitors trapped PARP1 and PARP2 to the sites of DNA damage and then the PARP enzyme-inhibitor complex locks onto the damaged DNA and stops DNA repair, transcription, and replication which then leads to cellular death [18]. Trapped PARP–DNA complexes were more cytotoxic than unrepaired single strand breaks (SSBs) caused by PARP inactivation, Murai et al. suggested that PARP inhibitors act in part as poisons that irreversibly trap PARP enzyme on DNA [19]. The potency in trapping PARP differed markedly among the PARP inhibitors in clinical development in a pattern not correlated with the catalytic inhibitory properties [20]. Thirty genetically altered avian DT40 cell lines with pre-established deletions in specific DNA repair genes were analyzed to reveal that, in addition to homologous recombination, post replication repair, the Fanconi anemia pathway, polymerase β, and FEN1 are critical for repairing trapped PARP–DNA complexes [18, 19]. This suggest that other defects in the HR pathway, including PTEN defects, Fanconi’s anemia protein defects, ATM abnormalities, RAD51 dysfunction, and EMSY defects, may be sensitive to single agent PARP inhibitors [20].

## PARP inhibitors

### Olaparib (Astra Zeneca)

The idea of synthetic lethality has led to the use of single agent PARP inhibitors in BRCA deficient cancers. Olaparib (AZD 2281) is an oral PARP inhibitor that has shown activity in ovarian and breast tumors with known *BRCA* mutations and was the first FDA approved drug in this class [21]. The first hint of clinical activity in *BRCA* mutation patients was seen in a phase I single agent trial with 50 ovarian cancer patients with *BRCA* mutations. Twenty patients had CR or PR by response evaluation criteria in solid tumors (RECIST) and three patients had been SD for longer than 4 months, resulting in a clinical benefit rate of 46 % (23/50). The median duration of response was 28 months. The most common drug related toxicities were fatigue and mild gastrointestinal (GI) symptoms. A post analysis showed a statistically significant difference in response among platinum sensitive, resistant, and refractory populations (61, 42, and 15 %, respectively), though no differences were noted in the duration of response or time to progression between the three platinum response groups [22]. The FDA approval of olaparib in advanced ovarian cancer associated with defective *BRCA* genes was partially based on an international multicenter single-arm trail with 317 patients whom 193 had measurable germline *BRCA* mutation ovarian cancer with a mean of 4.3 prior lines of therapy and considered platinum resistant. They were given 400 mg oral olaparib twice a day until progression or toxicity. They showed an overall response rate of rate of 31 % (95 % CI, 24.6 to 38.1), and stable disease (at 8 weeks) of 40 % (95 % CI, 33.4 to 47.7) along with a median OS of 16.6 months [23].

PARP-1/2 inhibitors have been demonstrated to be effective in preclinical models in combination with platinum, alkylating and methylation agents, topoisomerase I inhibitors and radiation therapy [16]. In a phase I/Ib trial of olaparib with carboplatin in germline *BRCA* mutation breast/ovarian cancer patients with an expansion cohort treated with 400 mg twice a day D 1–7 along with carboplatin AUC 5 days one every 21 days followed by maintenance of olaparib 400 mg twice a day until progression showed an ORR of 52.4 %. Responses included one complete response (one breast cancer; 23 months) and 21 partial responses (50.0 %; 15 ovarian cancer; six breast cancer; median = 16 [4 to >45] in ovarian cancer and 10 [6 to >40] months in breast cancer) [24]. Olaparib was also combined with carboplatin and paclitaxel in a study aimed to determine the safety of olaparib in one of four dosing regimens: continuously with carboplatin, continuously with paclitaxel, continuously with both carboplatin and paclitaxel or intermittently with the chemotherapy combination. Eighty seven patients were enrolled, 12 of whom had known germline *BRCA* one or two mutations. AEs were primarily myelotoxicity (neutropenia and thrombocytopenia of any grade occurring in 54 and 26 %, respectively). The dosing schedules deemed tolerable were olaparib with weekly paclitaxel (100 mg BD continuously and 80 mg/m2, respectively) and intermittent olaparib with 3-weekly doses of carboplatin and paclitaxel (200 mg BD d1-10 and AUC4 with 175 mg/m2, respectively). Sixteen percent of patients had an objective response and 28 % had stable disease that persisted for at least 4 months. Greater efficacy was evident in patients with *BRCA* mutations (two complete and four partial responses) [25]. Oza conducted a randomized phase II study, comparing six cycles of carboplatin and paclitaxel with olaparib (olaparib 200 mg/m2 BID d1-10 & carboplatin AUC 4 D1 & paclitaxel 175 mg/m2 D1, over a 21 day cycle) followed by maintenance olaparib (400 mg BID) until progression to six cycles of carboplatin and paclitaxel alone (AUC 6 and 175 mg/m2 respectively both D1, over a 21 days cycle), in patients with advanced serous ovarian cancer. The primary outcome, progression free survival, significantly favored those patients receiving olaparib in addition to chemotherapy (HR = 0.51, 95 % *P* = 0.0012) increasing median survival from 9.6 to 12.2 months [26].

Olaparib with cisplatin and gemcitabine was evaluated as a phase I trial by Rajan in advanced solid tumors. They saw high rates of myelosuppression even at early dose levels (DL1 olaparib 100 mg orally BID D1-4, gemcitabine 500 mg/m2 D3 & 10, and cisplatin 60 mg/m2 D3) which prompted dose reductions. Of the 21 patients which they evaluated two had PR. MTD was determined to be olaparib 100 mg orally once daily on D1, gemcitabine 500 mg/m2 on D1 & 8, and cisplatin 60 mg/m2 on D1. They were also able to demonstrate the olaparib inhibited PARP in PBMC and tumor tissue although they said that PARP levels were less efficiently inhibited when it was used for a short duration based on their observations that maximum inhibition of PAR was seen between 6 and 24 h after the first dose of administration and that PAR levels had started approaching baseline values within 36 h of the last dose of olaparib and exceeded baseline values in 80 % of cases before the next cycle of treatment [27]. There are a number of ongoing Phase I and II trials which various combinations currently underway and should have some promising results based on early phase trials.

After a number of early trials help to solidify the mechanism of action, the idea of maintenance therapy was explored. A randomized, double-blind, placebo-controlled study evaluated maintenance treatment with olaparib in patients with platinum-sensitive, relapsed, high-grade serous ovarian cancer who had received two or more platinum-based regimens and had had a partial or complete response to their most recent platinum-based regimen. Two hundred sixty-five patients were randomized 1:1 to 400 mg bid of olaparib vs placebo. Their primary end point was progression-free survival. Progression-free survival was significantly longer with olaparib than with placebo (median, 8.4 months vs. 4.8 months from randomization at time of completion of chemotherapy; hazard ratio for progression or death, 0.35; 95 % confidence interval [CI], 0.25 to 0.49; *P* < 0.001) Subgroup analyses of progression-free survival showed that, regardless of subgroup, patients in the olaparib group had a lower risk of progression. The first interim analysis of overall survival (38 % maturity) showed no significant difference between groups (hazard ratio with olaparib, 0.94; 95 % CI, 0.63 to 1.39; *P* = 0.75) [28]. At the second interim analysis, subgroup analysis was included. *BRCA* status was known for 131 (96 %) patients in the olaparib group versus 123 (95 %) in the placebo group, of whom 74 (56 %) versus 62 (50 %) had a deleterious or suspected deleterious germline or tumor *BRCA* mutation. Of patients with a *BRCA* mutation, median PFS was significantly longer in the olaparib group than in the placebo group (11.2 months [95 % CI 8.3-not calculable] vs 4.3 months [3.0–5.4]; HR 0.18 [0.10–0.31]; *p* < 0.0001); similar findings were noted for patients with wild-type *BRCA*, although the difference between treated and placebo groups was lower (7.4 months [5.5–10.3] vs 5.5 months [3.7–5.6]; HR 0.54 [0.34–0.85]; *p* = 0.0075). OS did not significantly differ between the groups (HR 0.88 [95 % CI 0.64–1.21]; *p* = 0.44); similar findings were noted for patients with mutated *BRCA* (HR 0.73 [0.45–1.17]; *p* = 0.19) and wild-type *BRCA* (HR 0.99 [0.63–1.55]; *p* = 0.96). The investigators concluded that these results support the hypothesis that patients with platinum-sensitive recurrent serous ovarian cancer with a *BRCA* mutation have the greatest likelihood of benefiting from olaparib maintenance therapy [29]. Moore presented two AstraZeneca-sponsored Phase III trials of olaparib maintenance monotherapy in ovarian cancer patients with a *BRCA* mutation: SOLO1 & SOLO2 at the 2014 ASCO meeting. Both are double-blind multicenter studies in which pts are being randomized (2:1) to receive olaparib (300 mg [2 × 150 mg tablets] bid) or placebo SOLO1 is for newly diagnosed patients and SOLO2 is for pretreated patients who have failed therapy. They have a planned analysis at ≈ 60 % maturity which is not available at time of writing.

Angiogenesis inhibitors have been shown to be active in recurrent ovarian cancer [30], and in vivo have been tested with PARP inhibitors. In PARP-1 knockout mice [31] the combination showed additive effects. Olaparib was looked at with cediranib in a phase I trial and appeared to improve PFS in women with recurrent platinum-sensitive high-grade serous or endometrioid ovarian cancer with hematologic DLT’s [32]. Sui looked at the combination of erlotinib and olaparib in EGFR-overexpressing ovarian tumor xenografts. They were able to show that erlotinib could slightly inhibit growth of A2780 tumor xenografts but the combination treatment had a markedly enhanced antitumor effect over either agent alone. They showed that the antitumor activity in BRCA-mutated xenograft models was 41 % compared with 24 % in BRCA wild-type. Western blot analysis revealed that treatment with erlotinib could significantly reduce the phosphorylation level of ERK1/2 and AKT in A2780 tumor tissue. It was shown that the autophagic effects were substantially enhanced when the agents were combined, which they postulated may be due to downregulation of apoptosis by decreasing p–p53 levels. Further investigations are underway to better understand these processes [33].

### Veliparib (abbvie)

Veliparib (ABT 888), in preclinical studies, was demonstrated to be a strong inhibitor of PARP 1 and 2 and was found to potentiate the effects of temozolomide, platinum agents, cyclophosphamide, and radiation in syngeneic and xenograft tumor models. It was reported to have good bioavailability and able to cross the blood–brain barrier [34]. Based on these broad spectrums of chemopotentiation and radiopotentiation further clinical evaluation was undertaken.

Veliparib was combined with oral cyclophosphamide in a phase II trial where adult patients with pretreated *BRCA*-mutant ovarian cancer or primary peritoneal, fallopian tube, or high-grade serous ovarian cancers (HGSOC). The patients were randomized to receive cyclophosphamide alone (50 mg orally once daily) or with veliparib (60 mg orally once daily) in 21-day cycles, crossover was allowed at disease progression. There were 75 patients enrolled with 72 evaluable, 38 cyclophosphamide alone and 37 on the combination arm. Of the 70 patients with responses one in each arm had a CR. PR was seen in six patients in the cyclophosphamide-only arm [7/36 (19.4 %) responses overall; 95 % confidence interval (CI), 8.2–36.0 %] and three patients in the combination arm [4/34 (11.8 %) responses overall; 95 % CI, 3.3–27.5 %], and one patient who crossed over to the combination arm after progressing on the cyclophosphamide-only arm. Overall the addition of veliparib to cyclophosphamide did not improve the response rate or the PFS over cyclophosphamide alone [35].

A phase II trial of veliparib was reported by Coleman et al., looking at the clinical activity with use as a single agent in ovarian cancer patients with a gBRCA1 or gBRCA2 mutation. The eligibility criteria included patients with three or fewer lines of therapy none of which would have been a PARPi. Veliparib was given at 400 mg orally twice a day for 28 day cycles. They reported response of 26 % (90 % CI: 16–38 %, CR: 2, PR: 11); for platinum-resistant and platinum-sensitive patients the proportion responding was 20 and 35 %, respectively. Overall 62 % were taken off study for progression, 29 patients were alive at the end of study; two with SD remained on veliparib and the median PFS reported was 8.18 months [36].

Recently Veliparib has also been evaluated with whole brain XRT for brain metastasis [37], combination with temozolomide in metastatic melanoma [38], small cell lung cancer with cisplatin and etoposide [39], as well as whole abdominal radiation for peritoneal carcinomatosis [40] all with promising results.

### Rucaparib (Clovis)

Rucaparib (AG014699) was initially studied as a first in class intravenous PARP inhibitor on an escalating dose design with temozolomide. There were 33 patients enrolled with PARP inhibition seen in PBMC at all doses through PK/PD studies with 74–97 % inhibition. The combination was well tolerated and there were encouraging responses in patients including one complete response and one partial response in melanoma, a partial response in a desmoid tumor, seven patients with prolonged disease stabilization (~6 months) [41]. The ongoing ARIEL 2/3 trails were presented at ASCO 2014 and consisted of two parts: ARIEL2 (NCT01891344), which is a Phase 2 trial of rucaparib trying to identify a molecular HRD signature which would predict response and Phase 3 ARIEL3 (NCT01968213), would then apply this signature prospectively to the analysis of a similar population. In ARIEL2, eligible patients (*n* = 180) who have relapsed, platinum-sensitive HGOC and measurable disease will have a pre-dose biopsy and provide archival tumor tissue. The design is then develop an initial HRD algorithm by using in vitro/in vivo and TCGA (and similar) bioinformatics data from Foundation Medicine’s NGS platform and Univ. of Washington’s BROCA-HR panel. The algorithm will be designed to correlate with tumor HRR status and PFS and response (RECIST v1.1, GCIG CA-125). Then prospectively in ARIEL3 (*n* = 540), optimized algorithm will then be tested an ongoing, randomized (2:1), placebo-controlled maintenance trial in platinum-sensitive HGSOC in remission after platinum-based therapy. The primary endpoint of ARIEL3 is PFS in HRD subgroups determined by NGS analysis of archival tumor tissue using the ARIEL2 optimized algorithm[42], these studies at time of manuscript writing are currently ongoing

### Niraparib (tesaro)

Niraparib (MK4827) is another oral inhibitor of PARP1 and PARP2. It was tested in phase I trial as a single agent in advanced solid tumors, ovarian tumors, and prostate tumors, and as combination therapy with carboplatin, with or without paclitaxel, and carboplatin with liposomal doxorubicin in patients with advanced solid tumors [43]. In a phase I trial of single agent niraparib enriched with patients having *BRCA*1 or *BRCA*2 mutations, six patients, including five with *BRCA* mutation, achieved PR [44]. Niraparib has also been shown to be an effective radiosensatizer especially in lung and breast cancer cells [44, 45]. Additionally Tesaro is currently sponsoring the Phase 2 QUADRA trial for patients with heavily pretreated disease [46] as well as the NOVA trial looking at maintenance in platinum sensitive disease [47].

### Talazoparib

Talazoparib (BMN 673) was designed as a potent novel inhibitor of PARP1 and PARP 2. Preclinical studies revealed selective antitumor cytotoxicity and causes expression of DNA repair biomarkers at much lower concentrations than that of earlier generations of PARP1/2 inhibitors [48]. Shen report that in vitro selectively targeted tumor cells with *BRCA*1, *BRCA*2, or PTEN gene defects with 20–to more than 200–fold greater potency than existing PARP1/2 inhibitors. BMN 673 is readily orally bioavailable and in vivo xenografted tumors that carry defects in *BRCA* or PTEN were profoundly sensitive to oral BMN 673 treatment. Synergistic or additive antitumor effects were also found when BMN 673 was combined with temozolomide, SN38, or platinum drugs in xenograft models. When evaluated in chicken DT40 cell lines, PALB2 mutation predicts exceptional in vivo response to BMN 673 [49]. Further studies showed that the nanomolar cytotoxicity is greater than that of rucaparib or olaparib and were believed to be related to the trapping of PARP-DNA complexes based on knockout mice models. All three drugs appeared to be equally effective at inhibiting PARP catalytic activity [50]. There are ongoing phase II trials in ovarian and phase III in breast cancer (EMBRACA) [51].

### Radiotherapy

PARP inhibitors enhance the effects of ionizing radiation by means of inhibiting base excision repair and non-homologous end joining as well as altered regulation of cellular metabolism. [52] Both pre-clinical and clinical data has shown an improvement in tumor response to irradiation in the presence of PARP inhibitors. It had been unclear if this benefit was due to changes in the repair process or vasoactive effects contributing to tumor re-oxygenation. The two questions that were asked was if in S-phase the PARP inhibition increased the radio-sensitivity of tumors and if at the tissue level it would affect the microvasculature [53]. Hirai, et al., looked at combination treatment with PARP inhibitors and single fraction gamma-irradiation and showed that treatment with a PARP inhibitor enhanced the cytotoxic effect of gamma-irradiation. PARP inhibitor treatment induced S phase arrest and enhanced subsequent G2/M arrest after irradiation. These results suggest that the induction of S phase arrest through an enhanced DNA Damage Response (DDR) and a local delay in dsDNA break processing by PARP inhibition caused sensitization to irradiation [54].

### Mechanisms of resistance

Multiple mechanisms of resistance to PARP inhibitors therapy have been identified. Intrinsic resistance to olaparib was show by increased up regulation of P-glycoprotein pumps in metaplastic breast carcinoma. This is a common pharmacological effect that reduces the efficacy of a number of drugs including PARP inhibitors by effluxing the drugs out of the cell and thus reducing the intracellular concentration of the drug available for the therapy [55]. Because PARP inhibitors can stabilize the cytotoxic PARP–DNA complexes, a loss-of-function of PARP1 can potentially lead to 100 fold resistance due to binding of PARP–DNA complexes and impaired catalytic inhibition of the PARP protein [56]. A mouse model resistant to olaparib showed up-regulation of a P-glycoprotein efflux pump caused by upregulating of ABCb1 a/b gene. The resistance can be reversed by inhibiting the pump with a P-glycoprotein inhibitor tariquidar [55]. Loss of 53BP1 leads to aberrant joining of complex chromosome rearrangements in Brca1-deficient cells by a process dependent on the non-homologous end-joining factors 53BP1 and DNA ligase 4. Loss of 53BP1 alleviates hypersensitivity of *BRCA*1 mutant cells to PARP inhibition and restores error-free repair by homologous recombination. 53BP1 deletion promotes ATM-dependent processing of broken DNA ends to produce recombinant single-stranded DNA competent for homologous recombination [57]. Another resistance mechanism to PARP inhibitor therapy that has been noted is via restoration of the homologous repair pathway in BRCA targeted tumors. BRCA2 mutant patients have shown resistance to PARP inhibitors by way of a secondary mutation in the BRCA2 gene that restores the open reading frame (ORF) which results in translation of a functional BRCA2 protein [58]. PARP inhibitor-resistant cells that up-regulation of NF-kappaB signaling is was suggested as a key mechanism underlying acquired resistance to PARP inhibition, and that NF-kappaB inhibition, or bortezomib are potentially effective anti-cancer agents after the acquisition of resistance to PARP inhibitors [59]. These are some of the mechanistic resistances to PARP inhibitors and more are being described as this class of drug continues to be studied.

### Immunotherapy

Advances in immunotherapy have been at the forefront of cancer development over the past few years with exciting developments showing significant benefits to patients. Combining DNA repair mechanisms with immune based therapy offer new frontiers in clinical advancements. Preclinical data exists for combining various PARPi with anti-CTLA-4, anti-PD1, as well as anti-PDL1 but there is little clinical data at this time. Higuchi looked at CTLA-4 blockade with PARPi ABT-888(Veliparib) in BRCA1-deficient murine ovarian cancer models and showed that combination CTLA-4/PARPi was able to provide therapeutic benefit in these experiments supporting further clinical investigations [60]. Trial NCT02571725 which is about to open will be looking at combining olaparib with tremelimumab in BRCA1/2 positive patients with recurrent ovarian cancer [61]. Trial NCT02484404 at the NCI is enrolling to look at novel anti-PDL1 (Durvalumab) in a Phase 1/2 in combination either with olaparib or cediranib initially in recurrent solid tumor but then in recurrent ovarian, with no data reported at this time [62]. At time of writing there is recent announcement of a trial about to open looking at combing niraparib and pembrolizumab in BRCA-positive breast and ovarian patients. Immune based therapies are breakthrough advancements in cancer care and combinations are appearing to offer promising results.

### Future directions

The approval of olaparib in the maintenance setting in Europe and metastatic setting in the US for patients with deleterious BRCA mutations in ovarian cancer is just the tip of the iceberg for the utilization for this class of agents. There are trials in progress to address the additional populations that may have deficiencies in the HR pathway that will benefit from PARP inhibitors. Additionally, combination trials with chemotherapy, radiation and TKIs are expanding the exploration of usage. Suggested by the cediranib and olaparib combination, combining PARP inhibitors with anther agent may not require additional DNA impairment for efficacy. Trials are also underway investigating agents that impair the DNA damage repair pathway, like veliparib and dinaciclib creating synthetic lethality without additional patient selection [63]. With greater understanding of resistance mechanisms, further trials utilizing combinations or sequential therapy to overcome the resistance to achieve greater efficacy. The duration of administration especially in the maintenance setting will also need to be considered to minimize resistance development. The current ongoing immune based combinations trials may bring additional synergistic efficacy and clinical benefit. This is a new class of agent that has endless possibilities for development and PARP inhibitors will be an important tool in the fight against cancer.

## Conclusions

PARP inhibitors is a new class of agents that have shown activity in ovarian cancer. Activity in non BRCA mutation related tumors are being explored both in ovarian as well as outside of ovarian cancer. New combinations with other targeted agents and immunotherapy will be areas of great interest in the next few years. 
